# Incremental diagnostic value of 18F-FDG PET/CT in the work up of adult limb and body wall sarcomas. A single centre experience

**DOI:** 10.1186/1470-7330-14-S1-P21

**Published:** 2014-10-09

**Authors:** N Ahmed, A Goldstone, G Avery, J Bates, AD Taylor

**Affiliations:** 1 Hull and East Yorkshire Hospitals NHS Trust, Hull, UK

## Background/aim

Soft tissue sarcomas (STS) amount to less than 1% of malignant adult tumours. There are more than 50 histological subtypes, some of which can be very aggressive and given their relative rarity, patients may pose unique diagnostic and therapeutic challenges. The aim of this exhibit is not only to share a single centre experience of the utility of [18F]FDG - PET/CT in these patients but also to offer pictorial demonstration of PET/CT appearances in some rare histological subtypes, which we feel will contribute towards the evolving role of PET/CT in this area.

## Patients and methods

Prospectively kept database for the sarcoma multidisciplinary meeting (MDM) was reviewed and patients undergoing 18F-FDG PET/CT examination at any stage of diagnostic workup for adult limb and body wall STS between 2011 and 2014 were reviewed. Patients with GIST and skeletal sarcoma were excluded. 17 patients were identified and the histology, FNCLCC grade, cross-sectional imaging including CT and MRI findings, the indication for performing PET/CT and the impact of its findings on MDM decision-making were evaluated.

## Results

PET/CT was utilised during initial pre-treatment staging in 10 and for assessing recurrent disease in 7 patients. The histology included 5 patients with leiomyosarcoma, 6 with various subtypes of liposarcoma, 2 with very rare extraskeletal osteosarcomas, 1 each of fibromyxoid sarcoma, solitary fibrous tumour, synovial sarcoma and undifferentiated sarcoma. By FNCLCC grading system, nine were grade 3, five were grade 2 and three patients had grade 1 tumours. PET/CT upstaged disease in 3/17 patients (identifying the true extent of local invasion in 1 patient and demonstrating subsequently proven metastases not detected on CT in 2 patients), refuted the suspicion of distant metastases on conventional imaging in 4/17, confirmed morphological findings in 5/17 and was inconclusive in 4/17 cases. In one patient with a chest wall leiomyosarcoma, a small paravertebral lesion was not evident on PET, likely due to its small size. High grade tumours (grades 2 and 3) were unequivocally hypermetabolic on PET, whilst grade 1 tumours and those with prominent a myxoid component were less FDG avid.

## Conclusion

In our single centre experience, PET/CT was found to be of significant incremental value in patients with high grade STS, by virtue of identifying occult foci of disease, detecting extent of loco-regional disease, and also by providing metabolic evidence in support or against equivocal findings on conventional imaging. This information contributed to confident decision making, particularly in patients in whom radical extremity amputations were under consideration.

